# The Role of Rehabilitation in Reducing Resource Use and Lowering Hospital Readmission Through Discharge Planning

**DOI:** 10.1093/geroni/igaa057.2919

**Published:** 2020-12-16

**Authors:** Kenneth Miller

**Affiliations:** UNT Health Science Center, Fort Worth, Texas, United States

## Abstract

The transitions between medical settings, the community and back again is a complex and intimidating process for patients, families and caregivers. These transitions are vulnerable points where planning is key and must begin at the initial examination with rehabilitation providers (PTs/OTs,SLPs). These providers are key members of the healthcare team to facilitate effective transition management. In this session, attendees will learn the critical factors rehabilitation providers use to evaluate patients in order to facilitate successful care transitions. An overview of the indications for rehabilitation referral will be presented, as well as evidence for effective rehabilitation strategies. The speaker will present tools from the American Physical Therapy Association Home Health Toolbox and outline a decision-making process for care transitions based on the individual, caregivers, and health care providers to achieve successful transitions that reduce resource use and hospital readmission rates. Attendees will learn strategies to facilitate inter-professional collaboration, communication, and advocacy.

